# Southern Surgical and Gynecological Association

**Published:** 1900-01

**Authors:** 


### *SOCIETY REPORTS.*

---

### SOUTHERN SURGICAL AND GYNECOLOGICAL ASSOCIATION.

*Proceedings of the Twelfth Annual Meeting, Held in New Orleans, La., December 5, 6, 7, 1899.*

FIRST DAY—MORNING SESSION.

The Association met at the St. Charles Hotel, and was called to order by the President, Dr. Joseph Taber Johnson, of Washington, D. C.

An address of welcome was delivered by Dr. Henry Dickson Burns on behalf of the local profession, which was responded to by President Johnson.

Dr. Ernest S. Lewis, Chairman of the Local Committee of Arrangements, made a brief report as to the work of the Association, entertainments, etc.

#### MYOMATOUS TUMOR OF EXTERNAL ILIAC VEIN WITH REPORT OF CASE.

Dr. A. M. Cartledge, Louisville, Ky., read the first paper. Myomatous tumors arising from the muscular coat of the veins constitute one of the rarest of pathologic curiosities, as a faithful search of the literature by the essayist discovered but two cases reported. A brief report of these was given. His own case occurred in a woman, aged fifty-three years. Five years ago she suffered from swelling of the left limb, extending from the foot to the hip; it came on gradually and the limb attained a considerable size; the swelling subsided in eight or ten months. She had pain at this time, in the left iliac region, low down. No enlargment was noticed. Two years ago she noticed a small lump just above Poupart's ligament on the left side from which she suffered some pain. Within the last year pain has been much greater in the left iliac region, enlargement more distinct and very hard; latterly she has been loosing much flesh, has slept poorly on account of pain, and has also suffered from accumulation of gas in the bowels. A diagnosis of carcinoma of the deep inguinal glands above Poupart's ligament was made, probably due to some primary, yet undiscoverable, carcinomatous lesion in the bowels or uterus. Exploration of the iliac tumor was advised for a more positive diagnosis, and removal, if found expedient. This was accepted and the operation performed on March 9, 1899.

Operation consisted of an incision beginning near the external abdominal ring, and running one inch above and parallel with Poupart's ligament, five inches in length. This came in contact with the tumor after opening the fascia of the external oblique muscle. The anterior part of the tumor occupied the inguinal canal; the round ligament was easily identified in front of the tumor, occupying the most fantastic relation to it, wound in and out around several lobules of the tumor; it was separated easily and pushed to the inner side. It was now seen that the tumor was not of glandular origin. By blunt dissection it was gradually freed from its upper and lower and external bed. The outer and deeper attachments were approached with great concern, as they were evidently in the closest relation with the external iliac vessels. Up to this time no vessels of importance had been encountered. It had been expected to run across the deep epigastric, but it nowhere appeared. Gentle and careful blunt dissection carried the pelvic peritoneum upward, no button-holing of the same occurring. After reaching the base and upper and outer part of the growth, the external iliac artery could be plainly felt entering the tumor. Search to the inner and posterior aspect of the artery failed to definitely demonstrate the vein: nothing more than a fibrous thickening could be made out, which was lost in the tumor substance. The point at which the external iliac artery entered the growth was about two inches above its lower termination. The apparent obliteration of the vein and its elimination as a factor in any surgical procedure affecting the circulation of the limb determined the essayist to ligate the artery one-half an inch above where it entered the growth. The fibrous remains representing the vein were similarly treated. The tumor was now separated from above downward, the deepest projection being disembedded from the obturator foramen; it now remained only attached to the vein and artery beneath Poupart's ligament. These were freed from the surrounding structures, by blunt dissection, and were ligated separately and the tumor removed. In cutting the vein below where it entered the growth, it was manifest that its lumen was almost obliterated, barely admitting a good-sized needle. The tumor was removed entire, with not a lobule broken, and passing through it and removed with it was one and three-fourth inches of the external iliac artery, and the same length of the nearly obliterated vein. The only hemorrhage of any account was from the obturator vein where it passes through the foramen; this was easily secured by ligation. The vessel was much enlarged, as a result of participating in a long-since-established collateral venous circulation. The upper portion of the incision was closed by tier sutures, the lower portion being left open, being occupied by a gauze drain which was placed down to the obturator foramen. The patient is progressing toward recovery.

Dr. William E. Parker, New Orleans, referred to the difficulty of diagnosticating tumors below the groin.

### URETERECTOMY.

Dr. J. Wesley Bovee, Washington, D. C., followed. He said that surgery of the kidney was practically of recent date; and surgery of the ureter was the logical sequence to renal surgery. The paper considered essentially the subject of ureterectomy, an operation less than nine years old, one seldom indicated, but offering brilliant results. Dr. Bovee reported the following case:

Mr. J. J. T., forty-eight years of age, was first seen with Dr. E. Reisinger, of Washington, in August, 1898. Three years previously his left kidney had been removed fór a renal abscess, and subsequently another operation had been done by the same surgeon for the remaining pus tracts and severe localized pain. He told the essayist that he had been an invalid constantly since before the first operation, and begged for relief. He had never been without pain and was frequently obliged to resort to morphia. He was much enfeebled, his pulse ranging from 110 to to 130, and his temperature from 99 to 101.6. On the left anterior and lateral aspects of the abdomen, slightly below the level of the umbilicus, were two scars of the previous operations. One was so near the median line that he was inclined to think the nephrectomy had been made transperitoneally. A large hernia had occurred at this site. There was three sinus tracts in the left lumbar region, discharging a thin, watery pus. In spite of the general weakness of the patient he decided an exploration to be advisable, and accordingly, on August 17, 1898, after free stimulation, he was anesthetized. With the assistance of Dr. Reisinger a careful exploration was made through an extraperitoneal incision, reaching from just above the level of the upper fistulous opening and in front of the left quadratus lumborum muscle to an inch inside the anterior superior iliac spine. By careful dissection and holding probes in the fistulæ the former location of the kidney was reached. Instead of the kidney a large amount of adipose tissue, containing many pus tracts and calculi and much cicatricial tissue, was found, and a little below it the upper end of the ureter surrounded by calculi and thickened pus. The ureter was about one inch in diameter and filled with cheesy pus and calculi. He resolved to remove the duct, and immediately extended the incision to the inguinal canal, keeping about one inch from Poupart's ligament. With considerable difficulty, but with very slight loss of blood, the ureter, in pieces, was removed to the bladder wall. The distended lumen ended abruptly about half an inch from the bladder wall, this portion being a solid cord. The wound was closed with through-and-through silkworm-gut sutures, a strip of iodoform gauze being passed to the lowest point of the pelvic wound and brought out at the lower end of the external incision, and another strip in the space formerly occupied by the kidney. The unusual atmospheric high temperature and humidity probably assisted in producing the fatal issue seventeen hours later.

Dr. Samuel E. Milliken, Dallas, Texas, called attention to a point in technique adopted by Kelly: Instead of making one incision, he leaves a bridge of abdominal wall, making the first incision from the twelfth rib down to the crest of the ilium in front of the lumbar muscles, with a second incision along the inguinal region. This enables the operator to expose the kidney, to make a thorough examination, and also to trace the ureter.

Dr. Bovee, in reply to Dr. Milliken, said there might be the advantage in two short incisions, of having a less amount of tissue to cut across, as muscles, fascia, etc., over one large one, but he thought there was a drawback in having to dissect through a smaller space to loosen the ureter between the two incisions. Such an operation would require more time, which, in many instances, is a precious element. However, he was not disposed to criticize Dr. Kelly's method, because he had never tried it.

SERIOUS COMPLICATIONS FOLLOWING PASSAGE OF URETHRAL SOUND.

Dr. William E. Parker, New Orleans, read a paper with this title. He reported a man with stricture of the urethra, who passed urine with difficulty. The first time a sound was passed he had a chill, with a temperature of 105. After a week the sound was passed again, with no fever having occurred between the days of the passage of the sound. In the meantime he had the man's urine examined, with a report showing the specific gravity to be 1020, .5 per cent. of albumin, no casts, pus and acid reaction. After the passage of the sound the second time the man had a urethral chill, suppression of urine, and died with uremic convulsions the following day, that is, the second day after the passage of the sound, although the usual precautions had been taken to prevent urethral chill. The autopsy showed that there was intense congestion of both kidneys, with pus in the pelvis, and in the calices of the left kidney.

Dr. William P. Nicolson, Atlanta, Ga., had seen a number of cases of severe chill following the introduction of sounds where all the necessary precautions had been taken; e. g., six cases of swelled testicle following the introduction of the sound, but had not seen a fatal case. In a book on the "Calamities of Surgery," Sir James Paget mentioned a case where the introduction of the catheter caused suppression of urine and death of the patient. The experience of genito-urinary surgeons would show, no matter how careful they were in sterilizing instruments, etc., in many cases, the apparent nervous connection between the organs would produce a severe chill and, in some cases, suppression of urine.

Dr. W. F. Parham, New Orleans, believed that post-mortem examinations would show, in cases where death had occurred after the passage of the urethral sound or catheter, some form of nephritic trouble, usually the interstitial. He recalled the case of a man on whom he operated for strangulated hernia, who had given no history of any renal trouble, yet died of interstitial nephritis, as verified by an autopsy.

Dr. Manning Simons, Charleston, S. C., had met with a number of cases in which the passage of the sound was followed by trouble. It is his routine practice, when a man presents himself for the first time, in whom he has to pass the sound, to administer 1 gr. of opium, and 10 gr. quinin immediately after its passage, as a prophylactic measure against urethral chill and fever. He thought the cases should be divided into three classes: 1, those in whom the trouble is reflex from the nervous system; 2, those in whom the urethritis is set up by unclean sounds; and, 3, those in whom there is serious disease of the kidney, but not discovered preceding the use of sounds.

Dr. J. D. Bloom, New Orleans, narrated two fatal cases from the passage of the sound, in New Orleans, and these showed kidney lesions post-mortem.

Dr. F. W. McRae, Atlanta, Ga., reported a fatal case which occurred in the practice of the elder Westmoreland, while the speaker was a medical student. He had seen complications follow the introduction of the sound. During the last five or six years he had not introduced a sound, except in the direst emergency, without having the patient under observation for several days thereafter. His results had been better since paying particular attention to careful preparation of the patient, instruments, repeated examinations of the urine, etc.

### GUNSHOT WOUNDS OF ABDOMEN.

Dr. H. H. Grant, Louisville, Ky., read this paper, which will be published in the Journal.

Dr. Hugh M. Taylor, Richmond, Va., said he had operated on three cases of gunshot wounds of the abdomen within the last two or three years. He laid stress on the utter hopelessness of cases until an operation was done. He was aware that arguments had been advanced by good men against surgical interference in many cases. It is not always easy to satisfy the attending physician, in the absence of serious symptoms, that an exploratory incision is imperative. Of the three cases narrated by him, one was saved.

Dr. William E. Parker was glad to note that the percentage of recoveries from operative interference in gunshot wounds of the abdomen is improving. Cases occurring in civil life should be operated on as soon as possible. In military life he has taken the stand that with the modern small-caliber bullet, as many cases will get well without operation in the field as with it. He mentioned a case operated on by the late Dr. Miles, in which there were fourteen intestinal perforations, which were closed, and the patient recovered.

Dr. Manning Simons had operated on twenty cases of gunshot wounds of the abdomen by laparotomy, and of this number he saved three. One was of the stomach, the operation having been done within a few hours after the occurrence. There were two perforations, one of the entrance, and one of the exit, the bullet having lodged in the muscles of the back. This patient, a boy, recovered and is well to-day. In one of the cases there were fifteen perforations of the bowel. In some he had resected as much as two feet of the small intestine.

Dr. Samuel E. Milliken had met with one case of gunshot wound of the abdomen in a child seven years of age, who had received the contents of a shotgun. He did a laparotomy, and the child recovered.

Dr. J. D. Bloom said the diagnosis of penetrating wound of the abdominal viscera was very essential in deciding on an operation. He mentioned the case of a man who was brought into the Charity Hospital, where it was thought the peritoneal cavity had been penetrated, but laparotomy disclosed that it had not. He recalled a number of patients who got well where the operation was done within twenty-four to thirty-six hours after being shot.

Dr. W. F. Parham said it was difficult in many instances to determine the exact location of the wound in the abdomen; therefore, the surgeon should study the character of the wound itself, its direction, the circumstances connected with the shooting, distance of the combatants, etc.

Dr. W. E. B. Davis, Birmingham, Ala., said the question to be settled was whether every case of penetrating wound of the abdomen should be operated on. He believes every such case should be so treated. The surgeon should carefully examine the wound of entrance so as to determine, if possible, whether penetration has taken place. He condemned the Senn gas test, and said that the teachings of this eminent surgeon in regard to gunshot wounds of the abdomen were harmful, in that they had created a sentiment which made it exceedingly difficult in certain communities to operate on cases of penetrating wounds of the abdomen, because, if the patient should die, the surgeon would be censured, and discredit cast on surgery. He and his brother (Dr. John D. S.) had operated on a number of cases of penetrating wounds of the abdomen from time to time.

Dr. F. W. McRae had seen and operated on five cases of gunshot wounds of the abdomen at the Grady Hospital. The point of entrance of the bullet was above the umbilicus, the injury having been received when the patient was standing, and in every one of them there were numerous perforations of the hollow viscera. In military practice, he said, wounds were received by soldiers who had been fasting for a number of hours, and the intestines were empty; extravasation was not so likely to occur. On the other hand, in civil practice wounds were almost always received under conditions of more or less debauchery, when the stomach was loaded with whisky, beer, food, etc., consequently extravasation of the contents took place rapidly, and death was much more apt to occur. He considered the Senn gas test worse than useless, it being merely corroborative, not conclusive as to penetration of the viscera.

Dr. Grant, in closing, said he had enunciated a few surgical principles, and he hoped the Fellows would conclude that it was the duty of the surgeon to operate as much in a penetrating wound of the abdomen as it was in cases of strangulated hernia.

### FIRST DAY—AFTERNOON SESSION.

### INTUBATION APPARATUS.

Dr. Rudolph Matas, New Orleans, presented a paper in which he gave the history of pulmonary insufflation and artificial respiration in intrathoracic surgery by intubation of the larynx, exhibited an apparatus, and demonstrated its application. In summing up the peculiarities of the apparatus he had shown he called attention to the following points: The original O'Dwyer canula, while retaining its intralaryngeal portion unchanged, is modified so that it may be utilized as a respirator, as a tampon canula, as an anesthetizer, as a tractor and tongue, as an insufflator, and as an aspirator.

### RECTOVAGINAL FISTULA.

Dr. Lewis S. McMurtry, Louisville, Ky., read a paper on this subject. He said that, excluding cancer extending from the cervix uteri, rectovaginal fistula is due to traumatism, not to the compression of the tissue, as in vesico-vaginal septum, cicatrizing inferiorly, but leaving a perforation above where the septum is thin and permits contact of vaginal and rectal mucous membranes, thus uniting these membranes and making a permanent opening. Wounds made in instrumentation through the vagina, and the pressure of foreign bodies retained in the vagina sufficiently long to produce ulceration and necrosis, are among the rare causes of this lesion.

The most common site is in the lower portion of the vagina, just above the sphincter muscles, and at the point already indicated where the septum is thin and the mucous surfaces are in close proximity one to the other. The opening is usually very small, and the mucous membrane is reflected around like a fringe, making its detection more amenable to touch than to sight.

The method usually applied to the repair of vesico-vaginal fistula, whereby the edges are freshened in a funnel-shaped denudation, will rarely succeed in repairing a rectovaginal fistula. The action of the anal sphincter, the penetration of fecal matter, and sparse layer of tissue prevent repair by this method. The divulsion of the sphincter muscle will not suffice to overcome this obstacle to success in the larger proportion of cases. Extensive cicatricial deposit is another obstacle to repair by this simple procedure. The operation in consequence is resolved into a modified perineorraphy, by which the fistula is transformed into a complete tear of the perineum. The method of flap-splitting popularized by the late Lawson Tait is preeminently applied to this procedure. By this method broad surfaces are supplied without loss of tissue, and permit gliding of the vaginal and rectal orifices of the fistula so as to interpose firm and healthy tissues. In exceptional cases, where the fistula is so low down as to be within the grasp of the sphincter, it will be best to divide the septum vertically, liberally freshening the margins of the fistula, and suture the surfaces as in complete laceration of the perineum.

In a very limited class of cases, wherein the fistula is small and cicatricial deposit is not extensive, a simple denudation and suturing after divulsion of the sphincter ani muscle may succeed. The flap-splitting method already mentioned will be found most applicable and should be preferred for general application. Vertical division of the septum should be the first step of the operation in low fistulæ with extensive cicatricial deposit, and the operation is then resolved, after paring the edges of the fistula, into the operation for complete tear of the perineum. It will rarely be necessary to use buried sutures; when used these should be of catgut and should be introduced after the Lembert plan. As already stated, the flap-splitting operation of Tait has the greatest field of usefulness in this procedure.

The following case is of special interest, both on account of the unique cause of the fistula, and the consequent deduction that very simple and common gynecological appliance is not without danger under certain circumstances.

Miss C. W., aged 31, never married, teacher, was the subject of a backward displacement of the uterus. In August, 1899, she applied to a physician for treatment, and a metallic pessary of the Hodge pattern was inserted. The pessary was placed on a Thursday by the physician who had referred the case to Dr. McMurtry, and the patient returned to her home, some thirty miles distant from the physician. On the second day the patient began to suffer pain, which increased day by day, and when examined by the physician on the Monday following the pessary was found protruding into the rectum and was removed per anum. When the patient was referred to him on October 6, there was an opening in the rectovaginal septum just above the anal sphincter, of oval form, that would readily admit the end of the little finger. The operation consisted of divulsion of sphincter, separation of the vaginal mucous membrane, freshening the edges of the fistula and suturing with Lembert sutures of catgut the rectal portion of the fistula, gliding and suturing the vaginal mucous membrane. Union was prompt and perfect, and the patient is now quite well.

In the discussion, cases of recto-vaginal fistula were reported by Drs. T. J. Crofford of Memphis, Tenn.; George Ben Johnston of Richmond, Va.; George H. Noble of Atlanta, Ga.; W. D. Haggard, Jr., of Nashville, Tenn., and J. Wesley Bovee of Washington, D. C.

### OSSIFIED UTERUS.

Dr. C. Jeff Miller of New Orleans, read the report of an interesting case of what he and others considered an ossified uterus. He presented the specimen.

### SECOND DAY—MORNING SESSION.

Dr. W. D. Haggard, of Nashville, Tenn., contributed a paper on

### THE SURGERY OF BILIARY CALCULI.

He emphasized the fact of the frequent finding of gall-stones in the abdominal cavity, but said their unsuspected existence was not a valid contraindication to operation in cases which, owing to certain complications, menace well-being and perhaps life itself. After enumerating the usually assigned causes of gall-stones, he impressed the undetermined probability of infection. The continuous pain in inflammatory and suppurative conditions of the biliary passages was contrasted with the intermittent and paroxysmal pain from biliary concretions. In connection with the diagnosis, the fact that jaundice was not essentially a sign of gall-stones was emphasized, and waiting for icterus as an evidence of their existence was hurtful.

The relatively small number of operations, as compared with the frequency of the disease, indicated that they were either overlooked, failed to give rise to serious trouble, or that the symptoms were taken for other diseases. Surgery of the gall-bladder was almost perfected, but the management of calculi in the ducts by various methods was yet unsettled.

The following case of cholo-cholecystotomy for chronic catarrhal cholangitis with gall-stones was reported:

B. J. G., white, male, aged twenty-seven years. He had his first severe attack of biliary colic attended with tenderness and jaundice in February, 1897. He had had a severe light attack of hepatic colic some weeks before. He was operated on the day after he was taken with the severe attack by Dr. J. F. W. Ross, of Toronto. A number of stones were removed, a fistula made, and a drainage-tube kept in for two weeks. The fistula remained open until June, 1897, when Dr. Ross closed it by suture, and the man, who had been in bed the whole of four month, was up in a week. In November, 1897, he again had a number of attacks of colic and jaundice, which kept up intermittently until March, 1898, when Dr. L. McLane Tiffany, of Baltimore, opened the gall-bladder, but finding no stones he closed it by immediate suture, fixing it to the abdominal wall, and closing the incision throughout. In June of the same year, while still in the hospital, he was again seized with attacks of colic and transient jaundice, which, in the absence of Dr. Tiffany in Europe, induced his assistant, Dr. I. R. Trimble, to again open the gall-bladder for exploration. Dr. Trimble found no stones, and thought he succeeded in passing a rubber catheter through the cystic duct, and thence into the duodenum. The gall-bladder was sutured and attached to the abdominal incision, which was closed by buried and superficial silkworm-gut sutures. On July 3d he had received a blow on the skull with a piece of wood in the hands of a drunken assailant, which resulted in a fracture of the skull, which was elevated by Dr. Robert Pillow, of Columbia, Tenn.

In August, 1899, fourteen months after the third gall-bladder operation, the pain and colic returned, and were rather frequent and more severe than ever before. On October 1st he had a spell that lasted four days, and could obtain no relief from anything. He came to see Dr. Haggard in Nashville, and while there had one of the worst attacks he had ever seen of biliary colic. He gave the patient $2\frac{3}{4}$ grains of morphine hypodermically in less than three hours, without any appreciable effect on the agonizing pain. He was not accustomed to taking morphine, and $\frac{1}{8}$ grain after the operation had a very happy and full effect.

On November 12, 1899, in the presence of jaundice, which had existed pitifully for two months, Dr. Haggard made an exploratory operation through the old scar parallel with the ribs. As the gall-bladder was attached to the parietes it was opened, and a black, tarry fluid was found therein, and one single, large gall-stone, soft and perfectly black, and somewhat larger than a cherry-stone, together with some smaller crystal-like stones. An effort was made to catheterize the ducts, as had been reported to have been done before without success. The gall-bladder was irrigated and packed with gauze temporarily. It was then dissected from the parietes below, and a careful palpation of the ducts was made, but no other stones could be detected. The adhesions were considerable, as the result of so many previous operations and such long-continued inflammatory trouble, and the gall-bladder was much contracted. Kelly, Senn and Murphy had all advised a cholecystectomy, but after he found no calculus obstruction to account for the recent colic and existing jaundice, he concluded it must be a chronic catarrhal cholangitis with inflammatory obstruction that came and went. He therefore deemed it unwise to do a cholecystectomy, and decided to do a cholecystenterostomy. The duodenum, however, was so matted with adhesions and the gall-bladder was contracted to such an extent that he did not think he could make the anastomosis with safety. He therefore utilized the hepatic flexure, and made a Murphy-button anastomosis with gauze drainage. There was no untoward symptom: a little bile came out after the gauze, when it was removed on the third day, but none thereafter, the drainage-tract closing quickly. The jaundice faded, the urine cleared up, and the patient had two normal bowel actions a day, whereas he had been previously taking purgatives daily. He went home at the end of two weeks with a clear complexion and a gain in weight, and has had no trouble since, but the button has not passed. McGuire reports a button retained over a year in cholecystenterostomy, but Treves has never had one remain in the gall-bladder.

In the discussion Dr. F. W. McRae narrated a case operated upon a few months ago, in which a diagnosis of cholecystitis was made. At the operation an unusual condition of affairs was found. Instead of a distended gall-bladder, he found enlargement of the liver, with great distention of a cavity in this organ, hard and nodular to the feel, and occupying this cavity and some small pockets in various directions in the liver were fifty-eight stones, which he exhibited. The adhesions were extensive, but no pus was found.

Dr. J. G. Earnest, of Atlanta, directed attention to the symptom jaundice, saying that it does not appear unless there is obstruction of the common duct. Simple obstruction of the cystic duct does not necessarily produce jaundice. In two or three cases in which he has found the gall-bladder filled with inspissated mucus and complete obstruction of the cystic duct but no obstruction of the common duct, there was no jaundice, while in other instances of common duct obstruction jaundice was present.

Dr. Hugh M. Taylor, of Richmond, Va., recalled a case which was diagnosticated at first as cholecystitis. The patient had been jaundiced for six weeks. The abdomen was opened, and on incising the gall-bladder and removing a stone from it he found no obstruction of either the cystic or common duct. He did a cholecystostomy, drained the gall-bladder, but the patient did not recover health. Two months later he reopened the abdomen, and, after a very careful examination, found malignant disease of the head of the pancreas sufficient to press upon the common duct and produce jaundice.

Dr. Manning Simons, of Charleston, S. C., reported a case in which he removed the whole gall-bladder, and said, after a careful search of literature at his command, he could find reports of only twenty cases. The tumor in his case presented just below the ninth rib as large as a goose egg. An exploratory operation was made, and the tumor was found to be the gall-bladder. The gall-bladder was removed, because its walls were greatly thickened and pus had disseminated itself throughout its walls. He believes that if the gall-bladder had not been removed at that time, and promptly, there would have been extravasation of pus into the abdominal cavity. Three gall-stones were found. There was very little jaundice present.

Dr. A. M. Cartledge, of Louisville, spoke of the cholemic cases, and said that in the use of the normal saline solution, employed subcutaneously or per rectum, for ten days, two weeks, or in bad cases, three weeks, before operative interference, surgeons had one of the most valuable means of overcoming cholemia in patients with gall-stones.

Dr. William P. Nicolson, of Atlanta, followed with a paper, entitled

### INFLAMMATION OF MECKEL'S DIVERTICULUM WITH RESULTING GANGRENE OF THE INTESTINE SIMULATING APPENDICITIS.

The pathological conditions arising from the diverticulum have been in some cases due to portions of the intestine being entrapped within the encircling cord where the diverticulum has existed in this form; while in some instances there has been an acute inflammation of the pervious tube that has caused adhesion and resulting obstruction. Again, the inflammation seems to have extended to the intestinal tube causing paresis of the bowel, especially simulating the later stages of appendicitis. In a few cases the symptoms and signs have been so much like inflammation of the appendix that a diagnosis has not been possible.

The author reported an interesting and instructive case, and mentioned briefly other cases he had found in consulting the literature. He also reported a case of successful implantation of an artificial testicle.

Dr. T. J. Crofford, of Memphis, reported

### TWO CASES OF INTRALIGAMENTOUS CYSTS.

CASE 1.—Miss T., aged fifty, presented herself with an abdominal tumor July 1, 1899. A few days later the abdomen was opened. The tumor was found to be an intraligamentous cyst. Both ovarian arteries were ligated; the uterine artery was ligated upon the healthy side at as low a level as the internal os. The uterus was cut across; the uterine artery secured upon the tumor side and the enucleation of the tumor proceeded with. The loss of blood was considerable, although the enucleation was done rapidly. Several arteries in the broad ligament required ligation, and one or two deep down in the pelvis needed to be secured before hemorrhage was under control. The cavity was obliterated as much as possible by suturing together the two layers of the ligament. The woman recovered.

CASE 2.—Mrs. S., aged forty-two, presented herself with an abdominal tumor October 31, 1899. During the month of June last she experienced an attack of acute peritonitis which came near terminating her life. The acuteness of the attack subsided, but a chronic peritonitis has existed ever since. Abdominal section was made November 6th. There were found two tumors, one on each side, developed between the layers of the broad ligaments. The ovarian arteries were secured near the brim of the pelvis. The tumors, which were not of very large size, were separated from the uterus as far down as the operator dared, and the uterine arteries were secured as low as possible after emptying the cysts. The hemorrhage was alarming at every attempt at enucleation upon all sides. The larger vessels in the broad ligaments and walls of the cysts, several in number, were, therefore, ligated. The upper portion of the sacs was trimmed off and covered with peritoneum. After the oozing had stopped the abdomen was closed without drainage. The patient has had an uninterrupted convalescence and has returned to her home.

The essayist said that Dr. Rufus B. Hall, of Cincinnati, had proposed the method of ligating both ovarian arteries and the uterine artery on the healthy side. The uterus was next amputated on a level with the internal os; the uterine artery on the tumor side was not secured, when he could peel out the cyst from between the layers of the broad ligament with a bloodless result. While he did not wish to detract from the merits of this achievement, yet the method is not to be implicitly relied upon as it will not control hemorrhage in all cases to a safe degree; that in some instances there will be found other vessels requiring ligation before the hemorrhage can be controlled within the bounds of safety. Some tumors of the broad ligament can be peeled out without hemorrhage; others will be attended with considerable loss of blood.

THE PRESIDENT'S ADDRESS.

This was delivered by Dr. Joseph Taber Johnson, of Washington, D. C. He touched on general subjects pertaining to the work and growth of the association. The Southern part of our country has furnished many noted surgeons and gynecologists to the medical profession, hence the name of the association derives greater appropriateness and significance from this fact. McDowell, Eve, Dudley, Sims, Thomas, Battey, and Emmet were referred to. The best gynecologists are now the best surgeons as far as they go. In abdominal, pelvic, and genital surgery, few general surgeons equal, and none surpass them. Their field of work was more narrow but none the less perfect and important on that account. While many gynecologists might take exception to such an absorption of their specialty, the gradual expansion of their work to the female pelvis gives the important argument for such an occurrence in the not distant future. It is not difficult to remember when gynecology was limited altogether to vaginal and pelvic work. Now, there are very few gynecologists who are not operating upon any tumor between the diaphragm and the vulva. The gynecologists attack ulcers and abscesses of the stomach and anastomose that organ with the intestine. They do all surgery of the ureter and kidney, the liver and gall-bladder, of the intestines, the spleen and pancreas. They operate for appendicitis and for all the varieties of hernia, and do the surgery of the bladder, the rectum, and the mammary gland.

There was no afternoon session of the second day. The members and guests took the steamer "Warren" and rode to the sugar plantation "Stanton," owned by Milliken and Ruttledge, and inspected it.

### SECOND DAY—EVENING SESSION.

Dr. Hugh M. Taylor, of Richmond, Va., read a paper on

### EXPERIENCE IN OPERATIONS FOR TYPHOID PERFORATION.

It is claimed that one-third of the deaths from typhoid fever are due to perforation. Typhoid perforation is credited with a mortality of 16,660 each year in the United States. The specialist in surgery appreciates what surgery can do and has done in the treatment of typhoid perforations. The general practitioner, in whose hands these cases annually first fall, is not so well informed, and he believes the association should express itself in no uncertain sound. It is claimed, too, that one-fifth of the deaths from typhoid fever are due to hemorrhage. This being so, it would seem just as unsurgical to let a typhoid patient die without an effort to save his life as it would be in the case of profuse hemorrhage from a gastric or duodenal ulcer, or even a ruptured tubal gestation. The author reported five cases, with one recovery from operation.

Less than two hundred cases have now been operated upon. When one recalled the thousands of cases annually occurring, the need for cooperative study is apparent. Nothing short of a moribund condition of the patient should warrant surgeons in abandoning a case as hopeless. The key to success is early operation. More than one fourth of the cases operated on have been saved by early surgical intervention. He believes that surgeons should be able to save more than 33½ per cent. by a timely operation.

Dr. F. W. McRae, of Atlanta, Ga., reported some interesting cases of abdominal surgery. The first case was one in which he operated for a pancreatic cyst; the second for hepatic calculi; the third for fecal fistulæ, and in the fourth case he did a combined appendectomy and nephrorrhaphy.

### LITHOLAPAXY.

Dr. George S. Brown, of Birmingham, Ala., read a paper with this title. A very limited experience with the operation of litholapaxy has led him to believe that it is being unwisely, although not altogether unreasonably, neglected. The most important cause for this neglect was that perhaps cutting operations, particularly the suprapubic, are very easy to do, and the results are very satisfactory. The author quoted the statistics of the different methods of operating for stone in the bladder, and reported cases in which he had resorted to litholapaxy with good results. The majority of stones met with in practice in this country can be removed with the lithotrite in the hands of an ordinarily careful surgeon, and that while it is not an operation to be undertaken by the general practitioner, no operating-room should be without one; suffering and time spent in bed are almost entirely eliminated, and the mortality is very much lower than that for either of the cutting operations.

### DECEMBER 7TH—THIRD DAY—MORNING SESSION.

Dr. E. D. Fenner, of New Orleans, La., reported fifteen cases of spinal injuries treated at the Louisiana Charity Hospital.

Dr. George H. Noble, of Atlanta, Ga., described the modification of an operation for cystocele. He also reported a case of seventeen years' congenital nocturnal incontinence of urine and one of pregnancy in a uterus bicornus.

Dr. I. L. Watkins, of Montgomery, Ala., presented a paper on "The Treatment of Retrodisplacements of the Uterus."

The following officers were elected: President, Dr. A. M. Cartledge, Louisville, Ky.; vice-presidents, Dr. Manning Simons, of Charleston, S. C., and Dr. W. P. Nicolson, of Atlanta, Ga.; secretary, Dr. W. E. B. Davis, of Birmingham, Ala.; treasurer, Dr. W. D. Haggard, Jr., of Nashville, Tenn. Atlanta, Ga., was selected as the place for holding the next annual meeting. The time for meeting was the second Tuesday in November, 1900.

